# Role of PET/CT in diagnosing and monitoring disease activity in rheumatoid arthritis: a review

**DOI:** 10.1007/s12149-023-01896-z

**Published:** 2024-01-26

**Authors:** Shashi B. Singh, Sambhawana Bhandari, Sadikshya Bhandari, Samikshya Bhandari, Rajshree Singh, William Y. Raynor, Soren Hess, Thomas J. Werner, Abass Alavi, Mona-Elisabeth Revheim

**Affiliations:** 1 Department of Radiology, Stanford University School of Medicine, Stanford, CA 94305 USA; 2 Danbury Hospital/Nuvance Health, Danbury, CT USA; 3 Nepal Medical College Teaching Hospital, Attarkhel, Kathmandu, Nepal; 4 Mercy Catholic Medical Center, Darby, PA 19023 USA; 5 Department of Radiology, Rutgers Robert Wood Johnson Medical School, 1 Robert Wood Johnson Place, MEB#404, New Brunswick, NJ 08901 USA; 6 Department of Nuclear Medicine, Odense University Hospital, Odense, Denmark; 7 Department of Radiology, Hospital of the University of Pennsylvania, Philadelphia, PA 19104 USA; 8 The Intervention Center, Division of Technology and Innovation, Oslo University Hospital, Rikshospitalet, Nydalen, Post Box 4950, 0424 Oslo, Norway; 9 Institute of Clinical Medicine, Faculty of Medicine, University of Oslo, Blindern, Post Box 1078, 0316 Oslo, Norway; 10 Department of Medicine, Division of Rheumatology, Washington University in St Louis, St Louis, MO USA; 11 Department of Clinical Research, Faculty of Health Sciences, University of Southern Denmark, Odense, Denmark

**Keywords:** Rheumatoid arthritis (RA), Positron emission tomography/computed tomography (PET/CT), [^18^F]-fluorodeoxyglucose ([^18^F]-FDG), [^18^F]-sodium fluoride ([^18^F]-NaF), Fibroblast activation protein inhibitor (FAPI), Diagnosis, Monitoring

## Abstract

Rheumatoid Arthritis (RA) is a systemic inflammatory disorder that commonly presents with polyarthritis but can have multisystemic involvement and complications, leading to increased morbidity and mortality. The diagnosis of RA continues to be challenging due to its varied clinical presentations. In this review article, we aim to determine the potential of PET/CT to assist in the diagnosis of RA and its complications, evaluate the therapeutic response to treatment, and predict RA remission. PET/CT has increasingly been used in the last decade to diagnose, monitor treatment response, predict remissions, and diagnose subclinical complications in RA. PET imaging with [^18^F]-fluorodeoxyglucose ([^18^F]-FDG) is the most commonly applied radiotracer in RA, but other tracers are also being studied. PET/CT with [^18^F]-FDG, [^18^F]-NaF, and other tracers might lead to early identification of RA and timely evidence-based clinical management, decreasing morbidity and mortality. Although PET/CT has been evolving as a promising tool for evaluating and managing RA, more evidence is required before incorporating PET/CT in the standard clinical management of RA.

## Introduction

Rheumatoid arthritis (RA) is a systemic inflammatory disorder resulting from immune dysregulation, which commonly presents as symmetric polyarthritis [1]. The prevalence of RA is estimated to be approximately 0.5–1.0% worldwide [2]. RA is most commonly seen in the age group 30–50, women, smokers, and those with a positive family history [3]. It may present with monoarthritis, oligoarthritis, or as systemic manifestations/complications. Usually, it presents with symmetrical joint pain, swelling of the small joints, and morning stiffness that lasts for more than an hour [4–6]. However, joint damage leading to permanent deformity can occur due to disease progression, especially if left untreated [4].

The systemic manifestations of RA include interstitial lung disease (ILD), pleural effusion, bronchiectasis, pericarditis, and various skin manifestations [1, 4, 7]. Patients may also present with symptoms of fatigue, weight loss, and anemia [3, 8]. Since RA can cause both articular and extra-articular complications, including rheumatoid nodules, rheumatoid vasculitis, pleuropulmonary, neurological, gastrointestinal, cardiovascular, cutaneous, hematologic, and ocular complications, international guidelines recommend starting therapy as soon as the diagnosis of RA is made [9, 10].

When a patient is suspected of having RA, a thorough medical history is taken with attention to joint pain, swelling, location of joints involved, duration of illness, and morning stiffness [3, 8, 11]. A complete physical examination is necessary to find joint involvement and other extra-articular features such as rheumatoid nodules [1]. Following this, blood tests with both rheumatoid factor (RF) and anti-citrullinated peptide/protein antibody (ACPA) testing have to be done [11]. Erythrocyte sedimentation rate (ESR) and serum C-reactive protein (CRP) are elevated in RA [1, 8]. Apart from these diagnostic tests, additional tests, including antinuclear antibody (ANA), complete blood count (CBC) with differentials, liver and renal function tests, and serum uric acid are performed in patients to exclude alternative diagnosis and to obtain a baseline value before initiating treatment [1, 12].

Imaging plays an important role in RA. X-rays are frequently employed to identify joint damage, but their sensitivity to early inflammatory changes is limited [13, 14]. Magnetic resonance imaging (MRI), distinguished for its heightened sensitivity to soft tissue alterations, proves valuable in early inflammation detection, although it may present constraints in terms of whole-body evaluation and is resource-demanding. Ultrasound facilitates real-time assessment of joint inflammation, though its efficacy may hinge on the operator's skill and is confined to superficial structures [15, 16]. Recently, molecular imaging modalities that can diagnose disease at an earlier stage and quantify the inflammation in rheumatoid arthritis and its myriad of complications over time are gaining attention. Positron emission tomography/computed tomography (PET/CT) with [^18^F]-FDG and other radiotracers is currently being studied to diagnose inflammatory states and to diagnose and monitor RA [17]. PET/CT distinguishes itself by detecting metabolic activity, providing a comprehensive, whole-body evaluation, and potentially revealing inflammation prior to observable structural changes in RA [18]. In this review article, we discuss the potential of PET/CT in diagnosing RA and its complications, evaluating the therapeutic response to treatment, and predicting RA remission.

## Evolving role of PET/CT in the evaluation of RA

PET was first introduced in the 1970s primarily to diagnose brain tumors [19]. However, the accumulation of [^18^F]-fluorodeoxyglucose ([^18^F]-FDG) at the sites of inflammation caused many false-positive oncological results [20, 21]. This increased uptake of [^18^F]-FDG is now the basis on which PET is used to diagnose and monitor inflammatory disorders, including RA [22]. When PET was combined with CT, a diagnostic modality that identified cellular metabolic information and the anatomical details of organs was developed [19].

**Fig. 1:**
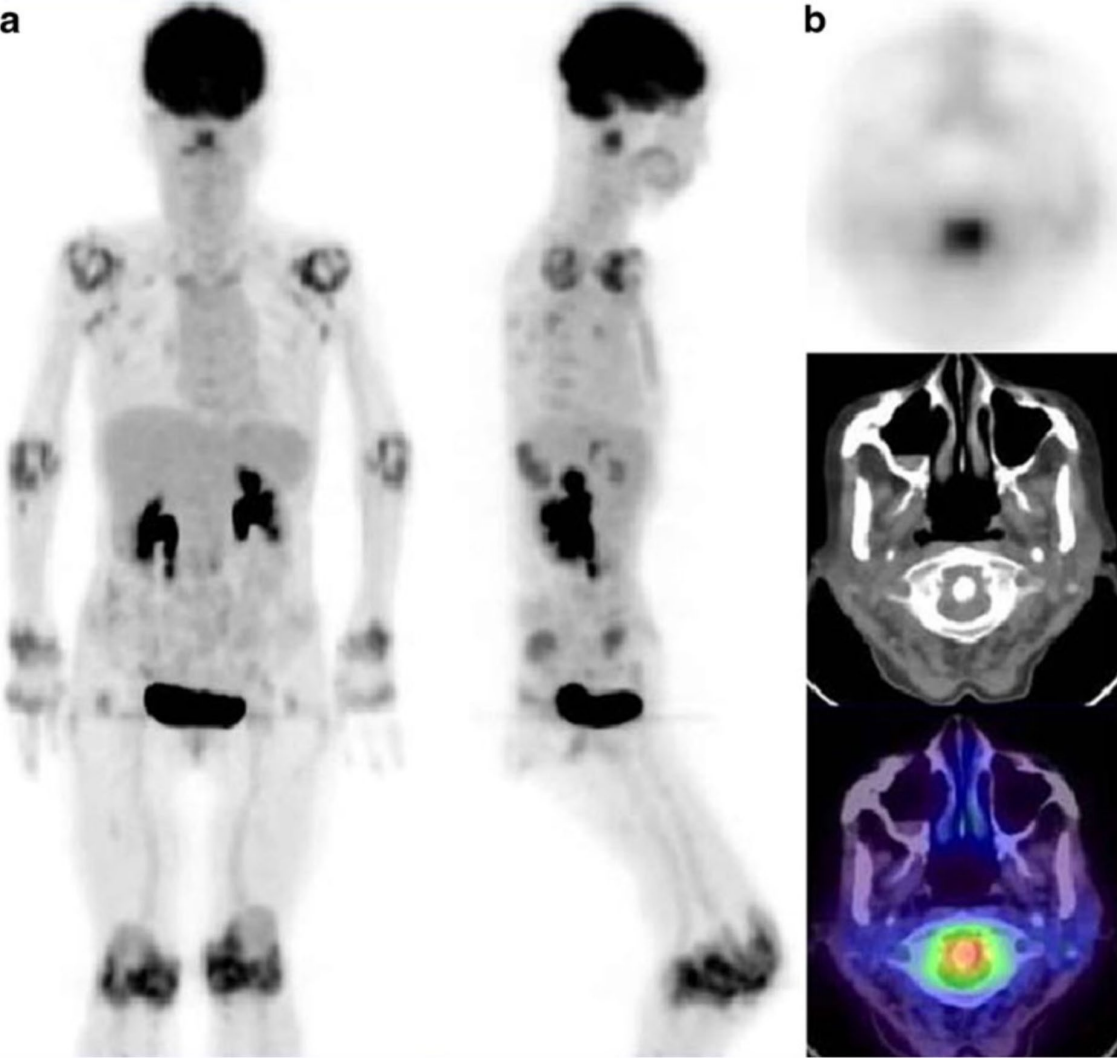
[^18^F]-FDG PET/CT was performed on a 71-year-old female with rheumatoid arthritis for seven years and was experiencing a flare-up. Maximum intensity projection (MIP) images of [^18^F]-FDG PET/CT in the anterior and right lateral views are shown in (**a**). Axial images of the atlantoaxial joint are shown in (**b**) (from top to bottom: PET, CT, and fused PET/CT). Atlantoaxial joint, right and left axillary lymph nodes, knees, hips, carpals, wrists, elbows, and shoulders all showed significant [^18^F]-FDG uptake. Reproduced with permission from [25]

In 1995, Palmar et al. reported the use of [^18^F]-FDG PET to quantify metabolic changes in the RA [23]. After this, multiple studies have studied its potential for diagnosing, predicting disease progression, monitoring disease activity, and therapeutic response to drugs Fig. 1 [23–25]. Studies have used [^18^F]-FDG PET to monitor signs of inflammation in the myocardium and wall of the blood vessels and to identify subclinical risk factors for cardiac complications in RA [26].

For more than 25 years, various PET parameters have been used to study different conditions. These include the maximum standardized uptake value (SUVmax) used for the quantification of inflammation, metabolic tumor volume (MTV), and total lesion glycolysis (TLG), which have been commonly used for the diagnosis of cancer [23]. For the assessment of RA, most studies have used SUVmax to evaluate the uptake of [^18^F]-FDG and other radiotracers at the sites of inflammation. It has been limited to a subjectively defined region of interest (ROI) [27] and, hence, does not represent the global disease activity in the patient, further increasing the importance of global disease score (GDS) in this context [28].

## Role of PET/CT in the evaluation of arthropathies in RA

PET/CT with [^18^F]-FDG and other radiotracers have been found to be promising for evaluating arthropathies and ruling out other differential diagnoses in RA. A study by Bhattarai et al. used the total visual score system to interpret [^18^F]-FDG PET findings in clinical settings. It evaluated commonly used PET parameters, including the total visual score, the total number of PET-positive joints, the sum of SUVmax, total metabolically active volume (MAV), TLG, and laterality bias in [^18^F]-FDG PET, to differentiate RA from other arthropathies. Visual evaluation with the total visual score and the total number of PET-positive joints was found to be the simplest tool to be used in practice and had a sensitivity of 88.9% and 94.9%, respectively [23].

Similarly, in another study by Raynor et al., SUVmax was able to characterize the activity of joint inflammation with [^18^F]-FDG PET, which was significantly different between RA and controls. Partial volume-corrected mean metabolic volume product (cMVPmean) calculated in a region delineated with a fixed threshold and representing volumetric and metabolic properties corrected for the partial volume effect showed a strong correlation to disease indicators, i.e., CRP, ESR, swollen joint count (SJC), Interleukin-6 (IL-6), IL-1, and Disease activity score (DAS) [27]. Similar results were observed in the study by Lee et al. in which PET-positive joints were found to be statistically correlated with SJC, tender joint count (TJC), and DAS28-ESR [29].

Although [^18^F]-FDG is one of the most commonly used radiotracers in assessing RA, other tracers are also being studied. [^18^F]-NaF PET/CT, useful in skeletal imaging, was found to have a strong positive correlation with DAS28-ESR and could accurately predict high disease activity in RA [30].

A systematic review published recently in 2022 discussed the potential of PET and SPECT imaging to target the biological pathways involved in angiogenesis in rheumatoid arthritis. It highlights the use of molecular imaging tracers to detect or monitor this angiogenic activity, with an emphasis on adhesion molecules like vascular adhesion protein-1 and integrins due to their roles in the angiogenesis [31]. Radiotracers limited to identifying synovial inflammation or angiogenesis as seen in the joints in RA could be helpful in the diagnosis of RA and differentiating it from other inflammatory arthritides [32]. Among them, RGD is a tri-peptide probe containing arginine, glycine, and aspartic acid, which targets alpha v beta 3 integrin, commonly expressed on the endothelium of angiogenic vessels. As neo-angiogenesis is a pathological mechanism of RA, it can be effectively used as a probe in PET/CT [33, 34]. Kavanal et al. studied this in 30 patients with RA, and increased tracer uptake was found in the synovium of the joints, tendon sheaths, and bursae [35]. Of the total 1560 joints examined, 394 were positive for increased synovial angiogenesis on [^68^Ga]-RGD2 PET/CT, while only 348 were positive on clinical evaluation. In 27 patients who underwent a follow-up after a median interval of 121 days, PET analysis showed good discrimination of non-responders from responders using SUVmax value at picking up non-responders with 100% specificity and responders with 86.4% specificity [35].

As the pathogenesis of RA involves the infiltration of synovium by macrophages, macrophage PET is another modality being studied in this context. PK11195(1-[2-chlorophenyl]-N-methyl-N-[1-methyl-propyl]-3-isoquinoline carboxamide) (*(R)*-[11C]PK11195) is a tracer that binds to the upregulated translocator protein (TSPO) in activated macrophages, and can visualize synovitis but has high background uptake in the periarticular tissues [17]. Verweij et al. conducted a study on 35 recently diagnosed RA patients. They underwent whole-body (*R*)-[11C] PK11195 PET/CT at baseline and in 2 weeks of COBRA (combination therapy of methotrexate and prednisone)-light treatment to predict the clinical response at 13 weeks [36]. Out of 1470 joints at baseline, 171 were positive on PET/CT, which decreased to 100 joints in 2 weeks. However, these changes in PET measures did not correlate with DAS44 at 13 weeks. However, the average SUV of the feet at 2 weeks significantly correlated with DAS44 at 13 weeks, predicting treatment response in the early RA [36].

Furthermore, two new probes, DPA-714 and DPA-713, which have an increased binding to TSPO and a less background uptake, have been developed [17, 37, 38]. Moreover, 110 joints in RA patients were studied to assess their specificity. In 80% of the joints, probe uptake was correlated to clinical signs, with higher uptake seen with [11C]DPA-713. Furthermore, background uptake was lower for both DPA tracers than *(R)*-[11C]PK11195 [17].

Besides diagnosis and monitoring, PET/CT can also be useful in ruling out differential diagnosis, for example, polymyalgia rheumatica (PMR). A study by Wang et al. found that SUVmax was the most valuable parameter in distinguishing RA from PMR [39]. It was lower in patients with RA than in PMR, with interspinous ligament showing the highest discriminative diagnostic value, with moderate sensitivity and high specificity [39]. In contrast, laboratory parameters, i.e., RF and anti-CCP antibodies, only achieved high sensitivity but had moderate specificity [39]. Additionally, other studies have demonstrated a notable increase in [^18^F]-FDG uptake in the ischial tuberosities, greater trochanters, and spinal processes among individuals with PMR as compared to those with RA [40, 41]. A systematic review and meta-analysis published recently has further explained the diagnostic utility of [18]-FDG PET/CT in PMR [42].

## Role of PET/CT in the evaluation of systemic complications of RA

Another important use of PET/CT in RA is to diagnose complications. Complications of RA commonly include but are not limited to vasculitis, rheumatoid nodules, respiratory complications (pleuritis, pleural effusion, ILD), cardiovascular diseases (myocarditis, coronary artery disease, and heart failure), neurological, nephrological, ocular, and hematological complications [4, 7, 9, 10]. Notably, with the recent advent of PET/CT, it has become possible to diagnose or even predict future complications of RA [43, 44].

### Cardiovascular complications

Cardiovascular disease (CVD) is one of the leading causes of death in RA secondary to vascular inflammation leading to atherosclerosis [45, 46]. Most assessments of atherosclerosis in RA to date have utilized imaging techniques that identify the presence of atherosclerotic plaque and luminal stenosis but not arterial wall inflammation [47]. Geraldino-Pardilla et al. studied 91 RA patients with [^18^F]-FDG PET/CT imaging of the ascending aorta. PET parameters were analyzed in comparison to other CVD risk factors and were found to be positively associated with each other [26]. HDL levels were inversely related to PET parameters [26]. The activity of RA disease measured by the DAS28-CRP and PET parameters of the ascending aorta was also found to vary in association with anti-CCP antibody levels [26]. Even in patients without any evidence of clinical cardiovascular disease, a PET scan at rest and with vasodilator stress has been used in a study to quantify myocardial blood flow (MBF) and myocardial flow reserve (MFR) using N-13 ammonia [48]. MFR was found to be lower in RA patients than in asymptomatic controls, with increased inflammation and higher mass and volume of the left ventricle [48]. However, the study could not predict if these changes increased the risk of heart failure or not [48].

**Fig. 2:**
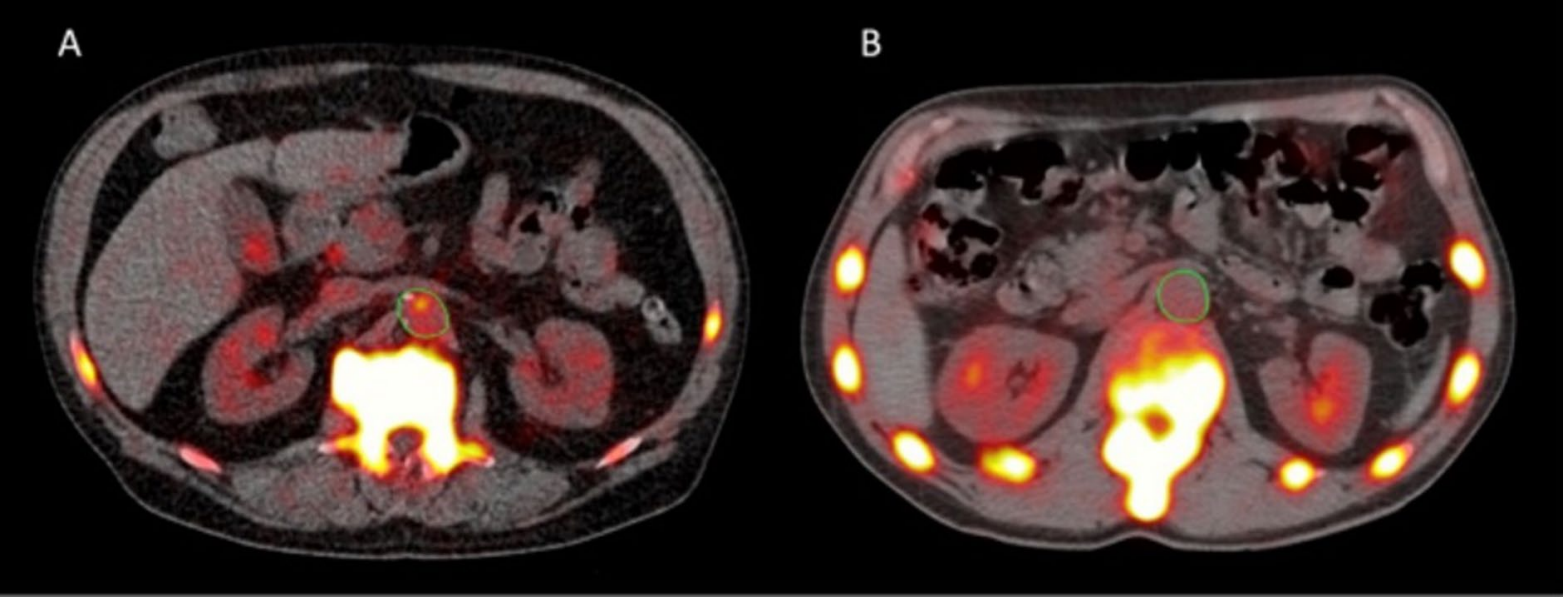
In this [^18^F]-NaF PET/CT study, regions of interest were manually outlined around the abdominal aorta, as shown in green. Comparison was made between the abdominal aortic wall of a 63-year-old patient with rheumatoid arthritis and an age- and sex-matched healthy control. The [^18^F]-NaF PET/CT scan revealed higher levels of [^18^F]-NaF activity in the abdominal aorta of the RA patient when compared to the healthy control. With permission from reference [20]

Moreover, [^18^F]-FDG PET/CT is being more commonly used to diagnose and evaluate atherosclerosis as [^18^F]-FDG can accumulate and show increased metabolism in the macrophages of the atherosclerotic plaque. Similarly, [^18^F]-FDG PET/CT can also be used to assess aortic inflammation in RA patients [43, 44]. High [^18^F]-FDG uptake was seen particularly in the walls of the carotid arteries and the aorta, which persisted after the values had been adjusted for traditional cardiovascular risk factors compared to patients with osteoarthritis [49]. It could also be one of the major pathogenesis for increased vascular complications in patients with RA [49]. Apart from [^18^F]-FDG PET/CT studies, assessment with [^18^F]-NaF PET/CT has also been found to be helpful in identifying increased microcalcification in the abdominal aorta in comparison to the healthy controls, which is predictive of atherosclerotic changes (Fig. 2) [20]. However, in this study, [^18^F]-FDG PET/CT was not able to identify any significant difference in microcalcifications between the RA and healthy control groups, contributing to its limitations [20].

### Pulmonary complications

**Fig. 3:**
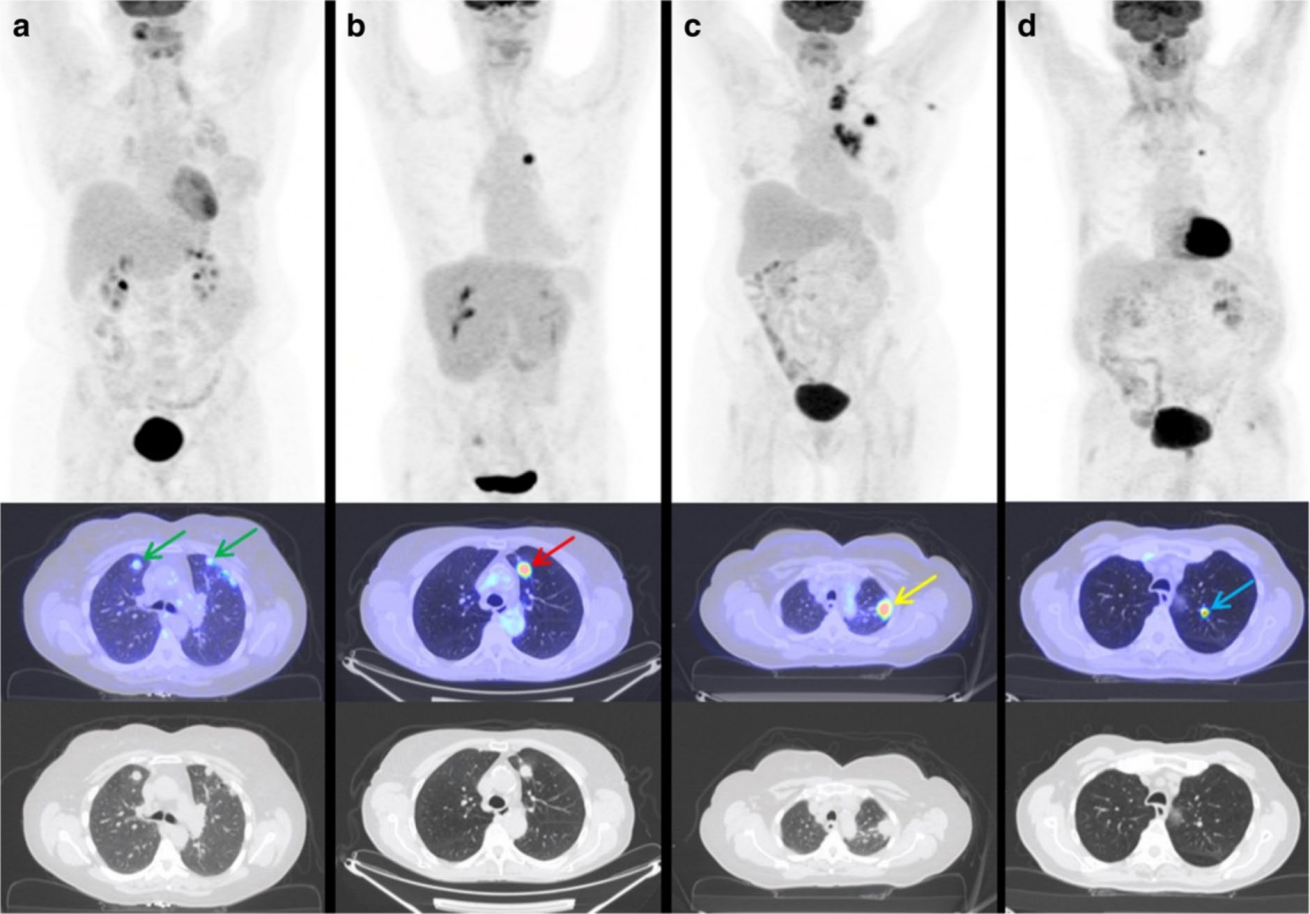
The above image demonstrates the PET/CT appearance of lung nodules, both benign and malignant, in patients with rheumatoid arthritis. The images were presented in three rows: the top row showed maximum intensity projection, the middle row showed fused PET/CT, and the bottom row showed CT scans. [^18^F]-FDG PET/CT images of rheumatoid arthritis patients with histologically proven rheumatoid nodules appeared as multiple, well-defined solid nodules located peripherally, with low [^18^F]-FDG activity (indicated by green arrows), in contrast to the appearances of squamous cell carcinoma (**b**), adenocarcinoma (**c**), and pulmonary carcinoid (**d**). With permission from reference [50]

RA can have several pulmonary manifestations, such as pulmonary parenchymal disease, including ILD, inflammation of the pleura (pleural thickening and effusions) and airways, and involvement of the pulmonary vasculature due to vasculitis, that can cause pulmonary hypertension [50]. PET/CT can detect inflammatory changes in the blood vessels due to vasculitis and lung parenchyma ranging from pulmonary nodules to ILD and resultant pulmonary fibrosis [50, 51]. As rheumatoid lung nodules can mimic cancer, it requires further diagnostic considerations. [^18^F]-FDG PET/CT has been seen to differentiate benign rheumatoid nodules from malignant nodules (Fig. 3) [50]. Although [^18^F]-FDG uptake may vary depending on the size and histology of lung cancer, posing challenges for differentiation in clinical settings, rheumatoid lung nodules exhibit a relatively low to moderate level of [^18^F]-FDG avidity and do not co-exist with [^18^F]-FDG-avid draining lymph nodes [50]. Additionally, since [^18^F]-FDG PET/CT scans the whole body, it may be possible to diagnose a lung lesion to be more likely to be a rheumatoid nodule than a lung cancer due to the presence of typical accumulations of [^18^F]-FDG in RA outside the lungs [52].

Likewise, uptake of [^18^F]-FDG is seen to be increased in both pathologically altered and normal appearing parenchyma of lungs in ILD [53, 54]. This uptake of [^18^F]-FDG in normal-appearing lung parenchyma is of prognostic value as it is found to be correlated to the severity of the disease [53, 55]. However, [^18^F]-FDG PET/CT has limitations in diagnosing and monitoring ILD as it lacks specificity [51]. Folate receptor (FR)-β, which is a type of glycosylphosphatidylinositol (GPI)-anchored protein and which usually binds folic acid and folate-linked molecules, is being studied as radiotracers [56, 57]. As FR-β has a high affinity with folic acid and internalizes them via endocytosis, they have been predicted to be useful. They are being used pre-clinically for imaging inflammatory conditions, including RA [51, 56, 57]. Currently, a new radiotracer based on [^18^F]-folate, 3′-Aza-2′-[^18^F]-fluoro-folic acid ([^18^F]-AzaFol), is being developed and has been predicted to be advantageous.

## Role of PET/CT in monitoring treatment response in RA

Despite many recent studies, clinical assessment of treatment response still requires a minimum of 12 weeks of RA [36]. [^18^F]-FDG PET can be used for systemic monitoring of disease. Amigues et al. studied eight patients in a nested longitudinal pilot sub-study who were escalated to tumor necrosis factor (TNF)-inhibitor or triple therapy (i.e., sulfasalazine + hydroxychloroquine with continued methotrexate). SUVmean was found to be 31% higher for those with a clinical disease activity index (CDAI) ≥ 10 than those with low scores, and SUVmean was 26% lower for non-TNF-targeted biologics than in non-biologic disease-modifying antirheumatic drugs (DMARDs). The myocardial SUVmean decreased 6 months after an escalation of therapy [58].

Similarly, Ravikanth et al. studied 42 RA patients undergoing anti-TNFα therapies and were assessed using whole-body [^18^F]-FDG PET/CT before and 3–6 months after therapy [59]. There was a correlation between ΔSUV and ΔDAS28 values measured six months after treatment with anti-TNF drugs. Thus, [^18^F]-FDG PET/CT was used to assess the distribution and extent of joint involvement in different phases of the disease, even in the same patient [59].

Another study was done on 15 RA patients refractory to anti-TNF-α treatments and was evaluated before and 16 and 24 weeks after rituximab administration. At baseline, there was a statistically significant correlation between PET by visual assessment and the SUVs to DAS28, CRP levels, and joint sonography results. However, at 16 weeks, this correlation was not seen. The PET/CT response was still found to be most accurate for predicting the clinical response at 24 weeks, with parameters individually showing an accuracy of 85.7% by visual assessment and 71.4% by the cumulative standard uptake value (cSUV) compared to only 64.3% by sonographic parameters and serum CRP values. However, PET/CT was not found to be useful in predicting rituximab response at week 24. It was concluded that [^18^F]-FDG PET/CT can be used to assess refractory RA and its response to therapy [24].

**Fig. 4:**
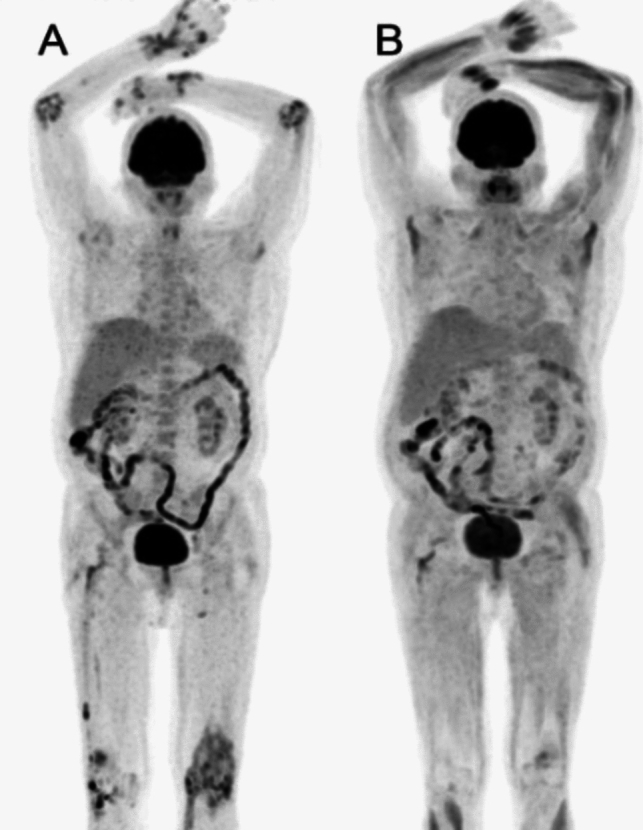
The study examined joint inflammation using [^18^F]-FDG PET/CT in a patient with rheumatoid arthritis at baseline (**A**) and after 12 months of tofacitinib treatment (**B**). The maximum intensity projection PET/CT image at baseline (A) displayed increased synovial activity in multiple joints, including the wrists, small hand joints, elbows, and knees bilaterally. However, in the follow-up image (**B**), after 12 months of tofacitinib treatment, there was a decrease in [^18^F]-FDG uptake, indicating a reduction in joint inflammation. With permission from reference [60]

**Fig. 5:**
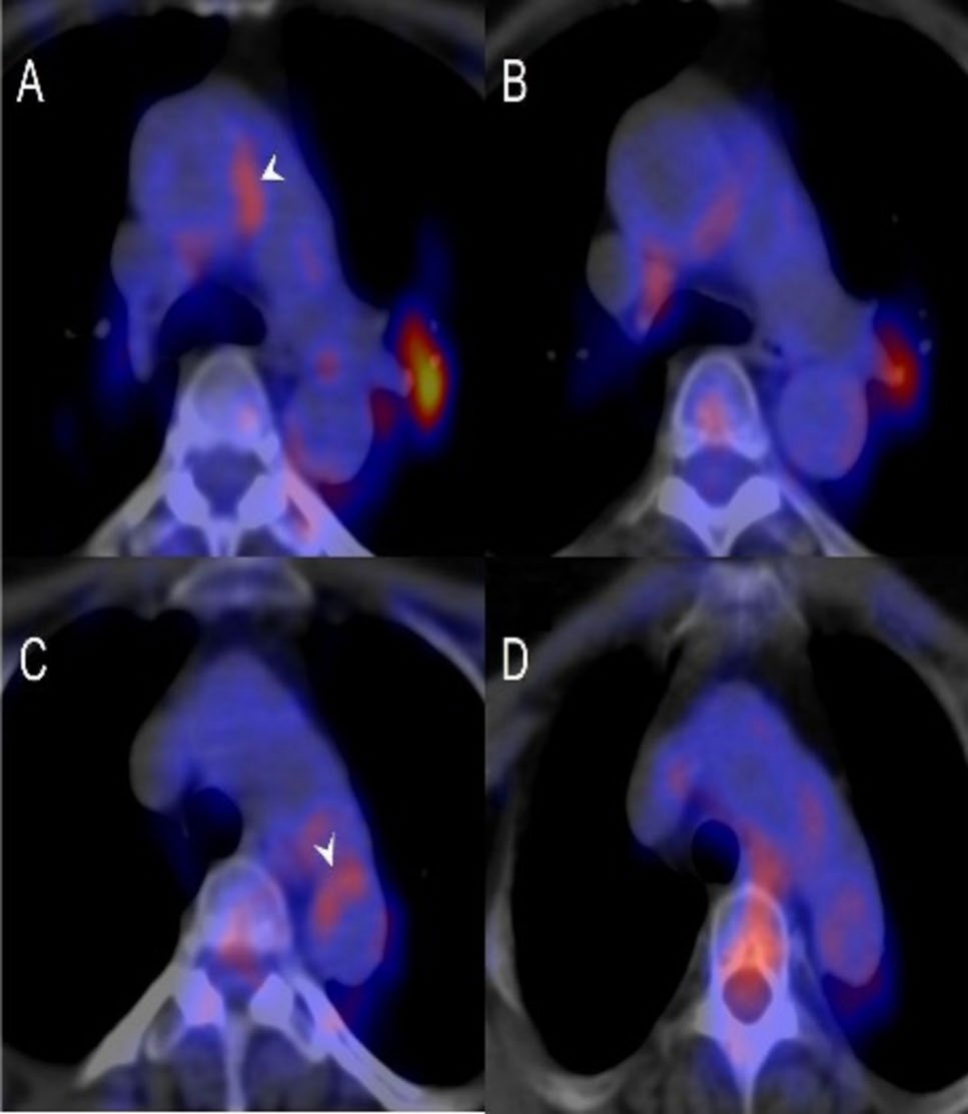
[^18^F]-FDG PET/CT images demonstrating aortic inflammation in patients with RA at baseline (**A**, **C**) and after therapy (**B**, **D**) with tofacitinib. With permission from reference [60]

[^18^F]-FDG PET/CT was also used to study changes following tofacitinib treatment at baseline and 12 months after therapy. It was shown that patients taking Tofacitinib had suppressed disease activity (as determined by PET/CT parameters: mean standardized uptake value-synovial (SUV-SYN_mean_) and mean target-to-background ratio-synovial (TBR-SYN_mean_) corroborated by ESR and CRP level (Fig. 4**)**. There was also improved aortic inflammation as measured by target-to-background ratio-vascular (TBR-VASCmax) (Fig. 5**)**. Therefore, this study has shown the appropriate use of [^18^F]-FDG PET/CT to assess both synovial and aortic inflammation for therapeutic drug monitoring in patients with RA [60].

In a study including 64 patients with RA treated with biologics for 6 months, they were assessed to compare inflammatory activity in the aortic walls [43]. The DAS28 and ESR were found to have significantly decreased after 6  months. However, there was little change in the [^18^F]-FDG uptake in the ascending aorta from baseline to 6 months, showing an SUVmax of 1.83 ± 0.34 to 1.90 ± 0.34 (*p* = 0.059) and a TBR of 1.71 ± 0.23 to 1.75 ± 0.24 (*p* = 0.222) [43]. It showed no significant decrease, thus predicting the limited use of biologics in preventing cardiovascular diseases [43]. However, another study, including 49 early but established RA cases treated with adalimumab, showed a favorable reduction in aortic wall inflammation after 6 months of treatment, thus again showing conflictive results [44].

Furthermore, 79 RA patients with low disease activity were assessed with [^18^F]-FDG PET/CT to apply PET parameters to predict the outcome of tapering TNF-inhibitor (TNFi) treatment for 18 months [61]. This study suggested that [^18^F]-FDG PET could detect clinical disease activity in patients with clinically low disease activity or remission. This study also suggested that a low resolution may have further affected the ability of PET to diagnose a low-activity joint signal. Thus, using the newest high-resolution digital PET scanners may show better and optimized results when assessing low-level inflammation in joints. However, [^18^F]-FDG PET parameters were not found to be predictive of tapering outcomes at 18 months [61].

Joint-draining lymph nodes (LN) are recently being assessed to predict the extent of joint inflammation and response to therapy in RA patients. Whole-body [^18^F]-FDG PET/CT can be used to assess these LN changes in a single scan [62, 63]. This was studied in 64 patients with involvement of the upper extremities in whom axillary lymph nodes were evaluated [62]. The ΔSUVmax values, i.e., activity in the axillary LN, were found to be significantly correlated with ΔDAS28-ESR, ΔDAS28-CRP, and serological markers in the patients. There was also a considerable decrease in ΔSUVmax value from 4.0 ± 2.6 to 2.3 ± 1.7 following 6 months of treatments with biological therapy, which corresponded to clinical and laboratory parameters [62]. Thus, the metabolic activity of axillary lymph nodes may serve as an indicator of the therapeutic response to biological therapy in patients with RA.

## Emerging evidence on the role of fibroblast activation protein inhibitor (FAPI) PET/CT in rheumatoid arthritis

Although [^18^F]-FDG PET/CT has been the mainstay of imaging patients with inflammatory disorders like RA, due to its limitations, an alternative radiotracer, possibly with theranostic potential, has always been sought. There is growing evidence for the usefulness of Fibroblast Activation Protein Inhibitor (FAPI) as a radiotracer for imaging RA [64–67]. Fibroblast-like synoviocytes (FLSs) are crucial effector cells in the inflamed joints of RA patients [68]. Previous research has shown that fibroblast activation protein (FAP) is abundantly expressed in RA-derived FLSs and serves as a unique marker for activated RA FLSs [69, 70]. Hence, attempts are being made not only to utilize FAPI to image RA FLSs but also to exploit its theranostic potential for the treatment of RA [65].

Ge et al. studied 1,4,7-triazacyclononane-N,N',N′′-triacetic acid-conjugated FAP inhibitor ([18F]AlF-NOTA-FAPI-04) for imaging RA FLSs in vitro as well as arthritic joints in RA patients [64]. The synovium of arthritic joints showed nonphysiologically high tracer uptake in RA patients who underwent [^18^F]AlF-NOTA-FAPI-04 PET/CT imaging. When compared to [^18^F]-FDG imaging, [^18^F]AlF-NOTA-FAPI-04 displayed a higher uptake in inflamed joints during the early stages of arthritis; moreover, this uptake was positively correlated with the arthritic scores further increasing its clinical importance. Hence, the authors concluded that [^18^F]AlF-NOTA-FAPI-04 is a promising radiotracer for imaging RA FLSs that may be used to supplement the existing noninvasive diagnostic criteria [64]. Similarly, a prospective study comparing the performance of gallium-68 ([^68^Ga])-labeled FAP inhibitor (FAPI) and [^18^F]-FDG for the evaluation of joint disease activity in RA showed that [^68^Ga]-FAPI demonstrates a greater amount and degree of affected joints than [^18^F]-FDG. Moreover, the extent of joint involvement on [^68^Ga]-FAPI PET/CT correlates with clinical and laboratory variables of the disease activity [66].

FAPI PET/CT could also be useful in evaluating pulmonary manifestations of RA [71]. Lung fibrosis induced by inflammation can be detected by FAPI [71]. FAPI PET/CT has been shown to detect both the presence and activity of lung fibrogenesis, making it a promising tool for assessing early disease activity and determining the efficacy of therapeutic interventions in patients with lung fibrosis [71]. Additionally, since FAPI has been useful in the detection of lung cancers [72], whether FAPI PET/CT holds the potential to differentiate benign rheumatoid nodules from malignant nodules needs to be explored further.

A case report demonstrated that [^68^Ga]-FAPI PET/CT might also be useful for seronegative RA [67]. As seronegative RA lacks the classical immunological markers, its clinical diagnosis is challenging. Cheung et al. presented [^68^Ga]-FAPI PET/CT results of seronegative RA in a 60-year-old woman and demonstrated how [^68^Ga]-FAPI PET/CT can aid in diagnosing seronegative RA [67].

The theranostic potential of FAPI has also given rise to an additional potential treatment modality for RA. A study demonstrates the application of FAP-targeted photodynamic treatment (FAP-tPDT) as a targeted locoregional therapy for RA [65]. In RA, activated synovial fibroblasts are crucial effector cells. The selective elimination of these cells based on their expression of fibroblast activation protein (FAP) is a promising therapeutic strategy. Dorst et al. came up with FAP imaging of inflamed joints utilizing [^68^Ga]-FAPI-04 PET/CT in a patient with RA and demonstrated the potential of selective anti-FAP-targeted photodynamic treatment (FAP-tPDT) in the synovium of RA patients ex vivo [65]. Nonetheless, further evidence is required to implement it in clinical settings.

## Conclusion

PET/CT seems to have significant potential as an imaging modality of choice in patients with RA and its myriad of complications. There are various studies reporting the use of PET/CT with [^18^F]-FDG, [^18^F]-NaF, FAPI, and other radiotracers for assessing the disease activity, therapeutic monitoring after drug use, escalation of therapy, tapering therapy, and identification and management of complications. It also has a role in ruling out other important differential diagnoses, including PMR. Although radiotracers claimed to have higher specificity in the evaluation of RA are recently being studied, their clinical usefulness is uncertain. Most are either in the pre-clinical phase or involve small sample-size studies with limited evidence. Thus, PET/CT with [^18^F]-FDG and [^18^F]-NaF has the potential to be a highly valuable diagnostic modality that can aid in the early diagnosis of RA and may help to guide intervention to limit its complications. However, further evidence is required before incorporating PET/CT in the routine clinical management of rheumatoid arthritis.

## References

[1] Sparks JA (2019). Rheumatoid arthritis. Ann Intern Med.

[2] Arima H, Koirala S, Nema K, Nakano M, Ito H, Poudel KM (2022). High prevalence of rheumatoid arthritis and its risk factors among Tibetan highlanders living in Tsarang, Mustang district of Nepal. J Physiol Anthropol.

[3] Wasserman A (2018). Rheumatoid arthritis: common questions about diagnosis and management. Am Fam Physician.

[4] Sparks JA, Barbhaiya M, Tedeschi SK, Leatherwood CL, Tabung FK, Speyer CB (2019). Inflammatory dietary pattern and risk of developing rheumatoid arthritis in women. Clin Rheumatol.

[5] Dong H, Julien PJ, Demoruelle MK, Deane KD, Weisman MH (2019). Interstitial lung abnormalities in patients with early rheumatoid arthritis: a pilot study evaluating prevalence and progression. Eur J Rheumatol Inflamm.

[6] Scherer HU, Häupl T, Burmester GR (2020). The etiology of rheumatoid arthritis. J Autoimmun.

[7] Lora V, Cerroni L, Cota C (2018). Skin manifestations of rheumatoid arthritis. G Ital Dermatol Venereol.

[8] Littlejohn EA, Monrad SU (2018). Early diagnosis and treatment of rheumatoid arthritis. Prim Care.

[9] Chen X, Zhang M, Wang T, Li Y, Wei M (2020). Influence factors of extra-articular manifestations in rheumatoid arthritis. Open Med.

[10] Smolen JS, Landewé R, Bijlsma J, Burmester G, Chatzidionysiou K, Dougados M (2017). EULAR recommendations for the management of rheumatoid arthritis with synthetic and biological disease-modifying antirheumatic drugs: 2016 update. Ann Rheum Dis.

[11] Aletaha D, Smolen JS (2018). Diagnosis and management of rheumatoid arthritis: a review. JAMA.

[12] Radu A-F, Bungau SG (2021). Management of rheumatoid arthritis: an overview. Cells.

[13] Fuchs HA, Kaye JJ, Callahan LF, Nance EP, Pincus T (1989). Evidence of significant radiographic damage in rheumatoid arthritis within the first 2 years of disease. J Rheumatol.

[14] van der Heijde DM, van Leeuwen MA, van Riel PL, Koster AM, van’t Hof MA, van Rijswijk MH (1992). Biannual radiographic assessments of hands and feet in a three-year prospective followup of patients with early rheumatoid arthritis. Arthritis Rheum.

[15] Ranganath VK, Hammer HB, McQueen FM (2020). Contemporary imaging of rheumatoid arthritis: clinical role of ultrasound and MRI. Best Pract Res Clin Rheumatol.

[16] Armstrong TM, Grainger AJ, Rowbotham E (2023). Imaging of rheumatological disorders. Magn Reson Imaging Clin N Am.

[17] Bruijnen STG, Verweij NJF, Gent YYJ, Huisman MC, Windhorst AD, Kassiou M (2019). Imaging disease activity of rheumatoid arthritis by macrophage targeting using second generation translocator protein positron emission tomography tracers. PLoS ONE.

[18] Chaudhari AJ, Raynor WY, Gholamrezanezhad A, Werner TJ, Rajapakse CS, Alavi A (2021). Total-body PET imaging of musculoskeletal disorders. PET Clin.

[19] Pijl JP, Nienhuis PH, Kwee TC, Glaudemans AWJM, Slart RHJA, Gormsen LC (2021). Limitations and pitfalls of FDG-PET/CT in infection and inflammation. Semin Nucl Med.

[20] Seraj SM, Raynor WY, Revheim M-E, Al-Zaghal A, Zadeh MZ, Arani LS (2020). Assessing the feasibility of NaF-PET/CT versus FDG-PET/CT to detect abdominal aortic calcification or inflammation in rheumatoid arthritis patients. Ann Nucl Med.

[21] Singh SB, Bhandari S, Siwakoti S, Bhatta R, Raynor WY, Werner TJ (2023). Is imaging bacteria with PET a realistic option or an illusion?. Diagnostics (Basel).

[22] Auletta S, Varani M, Horvat R, Galli F, Signore A, Hess S (2019). PET Radiopharmaceuticals for specific bacteria imaging: a systematic review. J Clin Med Res.

[23] Bhattarai A, Nakajima T, Sapkota S, Arisaka Y, Tokue A, Yonemoto Y (2017). Diagnostic value of 18F-fluorodeoxyglucose uptake parameters to differentiate rheumatoid arthritis from other types of arthritis. Medicine.

[24] Fosse P, Kaiser M-J, Namur G, de Seny D, Malaise MG, Hustinx R (2018). 18F- FDG PET/CT joint assessment of early therapeutic response in rheumatoid arthritis patients treated with rituximab. Eur J Hybrid Imaging.

[25] Carey K, Saboury B, Basu S, Brothers A, Ogdie A, Werner T (2011). Evolving role of FDG PET imaging in assessing joint disorders: a systematic review. Eur J Nucl Med Mol Imaging.

[26] Geraldino-Pardilla L, Zartoshti A, Bag Ozbek A, Giles JT, Weinberg R, Kinkhabwala M (2018). Arterial inflammation detected with 18 F-fluorodeoxyglucose-positron emission tomography in rheumatoid arthritis. Arthritis Rheumatol.

[27] Raynor WY, Jonnakuti VS, Zirakchian Zadeh M, Werner TJ, Cheng G, Zhuang H (2019). Comparison of methods of quantifying global synovial metabolic activity with FDG-PET/CT in rheumatoid arthritis. Int J Rheum Dis.

[28] Høilund-Carlsen PF, Edenbrandt L, Alavi A (2019). Global disease score (GDS) is the name of the game!. Eur J Nucl Med Mol Imaging.

[29] Lee SJ, Jeong JH, Lee C-H, Ahn B-C, Eun JS, Kim NR (2019). Development and validation of an 18 F-fluorodeoxyglucose-positron emission tomography with computed tomography-based tool for the evaluation of joint counts and disease activity in patients with rheumatoid arthritis. Arthritis Rheumatol.

[30] Park HJ, Chang SH, Lee JW, Lee SM (2021). Clinical utility of F-18 sodium fluoride PET/CT for estimating disease activity in patients with rheumatoid arthritis. Quant Imaging Med Surg.

[31] Khodadust F, Ezdoglian A, Steinz MM, van Beijnum JR, Zwezerijnen GJC, Jansen G (2022). Systematic review: targeted molecular imaging of angiogenesis and its mediators in rheumatoid arthritis. Int J Mol Sci.

[32] de Oliveira ÉA, Faintuch BL, Seo D, Barbezan AB, Funari A, Targino RC (2018). Radiolabeled GX1 peptide for tumor angiogenesis imaging. Appl Biochem Biotechnol.

[33] Leblond A, Allanore Y, Avouac J (2017). Targeting synovial neoangiogenesis in rheumatoid arthritis. Autoimmun Rev.

[34] Attipoe L, Chaabo K, Wajed J, Hassan F-U, Shivapatham D, Morrison M (2020). Imaging neoangiogenesis in rheumatoid arthritis (INIRA): whole-body synovial uptake of a 99mTc-labelled RGD peptide is highly correlated with power Doppler ultrasound. Ann Rheum Dis.

[35] Kavanal AJ, Bhattacharya A, Sharma A, Shukla J, Chattopadhyay A, Adarsh MB (2021). Prospective comparison of angiogenesis-specific 68Ga-RGD2 PET/CT imaging parameters and DAS28-ESR in rheumatoid arthritis. Clin Nucl Med.

[36] Verweij N, Zwezerijnen G, Ter Wee M, de Jongh J, Yaqub M, van Schaardenburg D (2022). Early prediction of treatment response in rheumatoid arthritis by quantitative macrophage PET. RMD Open.

[37] Dupont A-C, Largeau B, Santiago Ribeiro MJ, Guilloteau D, Tronel C, Arlicot N (2017). Translocator protein-18 kDa (TSPO) positron emission tomography (PET) imaging and its clinical impact in neurodegenerative diseases. Int J Mol Sci.

[38] Narayan N, Owen DR, Mandhair H, Smyth E, Carlucci F, Saleem A (2018). Translocator protein as an imaging marker of macrophage and stromal activation in rheumatoid arthritis pannus. J Nucl Med.

[39] Wang G, Liu X, Chen J, Zhang F, Xu X, Wang Y (2022). The Combination of 18F-fluorodeoxyglucose positron emission tomography metabolic and clinical parameters can effectively distinguish rheumatoid arthritis and polymyalgia rheumatic. Contrast Media Mol Imaging.

[40] Wakura D, Kotani T, Takeuchi T, Komori T, Yoshida S, Makino S (2016). Differentiation between polymyalgia rheumatica (PMR) and elderly-onset rheumatoid arthritis using 18F-fluorodeoxyglucose positron emission tomography/computed tomography: is enthesitis a new pathological lesion in PMR?. PLoS ONE.

[41] Takahashi H, Yamashita H, Kubota K, Miyata Y, Okasaki M, Morooka M (2015). Differences in fluorodeoxyglucose positron emission tomography/computed tomography findings between elderly onset rheumatoid arthritis and polymyalgia rheumatica. Mod Rheumatol.

[42] van der Geest KSM, Treglia G, Glaudemans AWJM, Brouwer E, Jamar F, Slart RHJA (2021). Diagnostic value of [18F]FDG-PET/CT in polymyalgia rheumatica: a systematic review and meta-analysis. Eur J Nucl Med Mol Imaging.

[43] Trang DAMT, Okamura K, Suto T, Sakane H, Yonemoto Y, Nakajima T (2021). Do biologic therapies reduce aortic inflammation in rheumatoid arthritis patients?. Arthritis Res Ther.

[44] Blanken AB, Agca R, van Sijl AM, Voskuyl AE, Boellaard R, Smulders YM (2021). Arterial wall inflammation in rheumatoid arthritis is reduced by anti-inflammatory treatment. Semin Arthritis Rheum.

[45] England BR, Thiele GM, Anderson DR, Mikuls TR (2018). Increased cardiovascular risk in rheumatoid arthritis: mechanisms and implications. BMJ.

[46] Majka DS, Vuha T-HT, Pope RM, Teodorescu M, Karlson EW, Liu K (2017). Association of rheumatoid factors with subclinical and clinical atherosclerosis in African American women: the multiethnic study of atherosclerosis. Arthritis Care Res.

[47] Chen J, Zhang X, Millican R, Sherwood J, Martin S, Jo H (2021). Recent advances in nanomaterials for therapy and diagnosis for atherosclerosis. Adv Drug Deliv Rev.

[48] Amigues I, Russo C, Giles JT, Tugcu A, Weinberg R, Bokhari S (2019). Myocardial microvascular dysfunction in rheumatoid arthritis: quantitation by 13N-ammonia positron emission tomography/computed tomography. Circ Cardiovasc Imaging.

[49] Agca R, Blanken AB, van Sijl AM, Smulders YM, Voskuyl AE, van der Laken C (2021). Arterial wall inflammation is increased in rheumatoid arthritis compared with osteoarthritis, as a marker of early atherosclerosis. Rheumatology.

[50] Koslow M, Young JR, Yi ES, Baqir M, Decker PA, Johnson GB (2019). Rheumatoid pulmonary nodules: clinical and imaging features compared with malignancy. Eur Radiol.

[51] Schniering J, Benešová M, Brunner M, Haller S, Cohrs S, Frauenfelder T (2019). 18F-AzaFol for detection of folate receptor-β positive macrophages in experimental interstitial lung disease-a proof-of-concept study. Front Immunol.

[52] Gupta P, Ponzo F, Kramer EL (2005). Fluorodeoxyglucose (FDG) uptake in pulmonary rheumatoid nodules. Clin Rheumatol.

[53] Bellando-Randone S, Tartarelli L, Cavigli E, Tofani L, Bruni C, Lepri G (2019). 18F-fluorodeoxyglucose positron-emission tomography/CT and lung involvement in systemic sclerosis. Ann Rheum Dis.

[54] Motegi S-I, Fujiwara C, Sekiguchi A, Hara K, Yamaguchi K, Maeno T (2019). Clinical value of 18 F-fluorodeoxyglucose positron emission tomography/computed tomography for interstitial lung disease and myositis in patients with dermatomyositis. J Dermatol.

[55] Hoffmann-Vold A-M, Distler O, De Vries-Bouwstra JK. Interplay between immunity and fibrosis [Internet]. Frontiers Media SA; 2021. Available from: https://play.google.com/store/books/details?id=QKhVEAAAQBAJ.

[56] Silvola JMU, Li X-G, Virta J, Marjamäki P, Liljenbäck H, Hytönen JP (2018). Aluminum fluoride-18 labeled folate enables in vivo detection of atherosclerotic plaque inflammation by positron emission tomography. Sci Rep.

[57] Chandrupatla DMSH, Jansen G, Mantel E, Low PS, Matsuyama T, Musters RP (2018). Imaging and methotrexate response monitoring of systemic inflammation in arthritic rats employing the macrophage PET tracer [18F]Fluoro-PEG-Folate. Contrast Media Mol Imaging.

[58] Amigues I, Tugcu A, Russo C, Giles JT, Morgenstein R, Zartoshti A (2019). Myocardial inflammation, measured using 18-fluorodeoxyglucose positron emission tomography with computed tomography, is associated with disease activity in rheumatoid arthritis. Arthritis Rheumatol.

[59] Ravikanth R, Singh JK (2020). Semi-quantitative analysis of 18F fluorodeoxyglucose uptake in the assessment of disease activity and therapeutic response in rheumatoid arthritis: an institutional experience. World J Nucl Med.

[60] Hamar A, Hascsi Z, Pusztai A, Czókolyová M, Végh E, Pethő Z (2021). Prospective, simultaneous assessment of joint and vascular inflammation by PET/CT in tofacitinib-treated patients with rheumatoid arthritis: associations with vascular and bone status. RMD Open.

[61] Bouman CAM, van Herwaarden N, Blanken AB, Van der Laken CJ, Gotthardt M, Oyen WJG (2022). 18F-FDG PET-CT in rheumatoid arthritis patients tapering TNFi: reliability, validity and predictive value. Rheumatology.

[62] Dam TT, Okamura K, Nakajima T, Yonemoto Y, Suto T, Arisaka Y (2020). Axillary lymph-node metabolic activity assessment on 18F-FDG-PET/CT in rheumatoid arthritis patients treated with biologic therapies. Scand J Rheumatol.

[63] Yamada C, Oguro E, Tsuji S, Kudo-Tanaka E, Teshigawara S, Ohshima S (2020). Pathological assessment of the lymph node biopsies for lymphadenopathy in rheumatoid arthritis. Mod Rheumatol.

[64] Ge L, Fu Z, Wei Y, Shi D, Geng Y, Fan H (2022). Preclinical evaluation and pilot clinical study of [18F]AlF-NOTA-FAPI-04 for PET imaging of rheumatoid arthritis. Eur J Nucl Med Mol Imaging.

[65] Dorst DN, Rijpkema M, Buitinga M, Walgreen B, Helsen MMA, Brennan E (2022). Targeting of fibroblast activation protein in rheumatoid arthritis patients: imaging and ex vivo photodynamic therapy. Rheumatology.

[66] Luo Y, Pan Q, Zhou Z, Li M, Wei Y, Jiang X (2023). 68Ga-FAPI PET/CT for rheumatoid arthritis: a prospective study. Radiology.

[67] Cheung SK, Chen S, Wong YH, Wu KK, Ho CL (2023). Diagnosis of seronegative rheumatoid arthritis by 68 Ga-FAPI PET/CT. Nucl Med Mol Imaging.

[68] You S, Koh JH, Leng L, Kim W-U, Bucala R (2018). Review: The tumor-like phenotype of rheumatoid synovium—molecular profiling and prospects for precision medicine. Arthritis Rheumatol.

[69] Bauer S, Jendro MC, Wadle A, Kleber S, Stenner F, Dinser R (2006). Fibroblast activation protein is expressed by rheumatoid myofibroblast-like synoviocytes. Arthritis Res Ther.

[70] Croft AP, Campos J, Jansen K, Turner JD, Marshall J, Attar M (2019). Distinct fibroblast subsets drive inflammation and damage in arthritis. Nature.

[71] Rosenkrans ZT, Massey CF, Bernau K, Ferreira CA, Jeffery JJ, Schulte JJ (2022). [68 Ga]Ga-FAPI-46 PET for non-invasive detection of pulmonary fibrosis disease activity. Eur J Nucl Med Mol Imaging.

[72] Wang L, Tang G, Hu K, Liu X, Zhou W, Li H (2022). Comparison of 68Ga-FAPI and 18F-FDG PET/CT in the evaluation of advanced lung cancer. Radiology.

